# Investigation of the relationship between phenylalanine in venous plasma and capillary blood using volumetric blood collection devices

**DOI:** 10.1002/jmd2.12398

**Published:** 2023-10-16

**Authors:** Rachel S. Carling, Zoe Barclay, Nathan Cantley, Erin C. Emmett, Sarah L. Hogg, Yael Finezilber, Danja Schulenburg‐Brand, Elaine Murphy, Stuart J. Moat

**Affiliations:** ^1^ GKT School Medical Education Kings College London London UK; ^2^ Biochemical Sciences, Synnovis, Guys & St Thomas' NHSFT London UK; ^3^ Department of Clinical Biochemistry, Severn Pathology Southmead Hospital, North Bristol NHS Trust Bristol UK; ^4^ Biochemical Genetics Unit Cambridge University Hospitals Cambridge UK; ^5^ Charles Dent Metabolic Unit National Hospital for Neurology and Neurosurgery, Queen Square London UK; ^6^ Department of Haematology, Immunology and Metabolic Medicine University Hospital Wales Cardiff UK; ^7^ Department of Medical Biochemistry, Immunology & Toxicology University Hospital Wales Cardiff UK; ^8^ School of Medicine Cardiff University, University Hospital Wales Cardiff UK

**Keywords:** DBS, dried blood spots, microsampling, phenylalanine, phenylketonuria, PKU, standardisation, volumetric blood collection devices

## Abstract

Measurement of plasma and dried blood spot (DBS) phenylalanine (Phe) is key to monitoring patients with phenylketonuria (PKU). The relationship between plasma and capillary DBS Phe concentrations has been investigated previously, however, differences in methodology, calibration approach and assumptions about the volume of blood in a DBS sub‐punch has complicated this. Volumetric blood collection devices (VBCDs) provide an opportunity to re‐evaluate this relationship. Paired venous and capillary samples were collected from patients with PKU (*n* = 51). Capillary blood was collected onto both conventional newborn screening (NBS) cards and VBCDs. Specimens were analysed by liquid‐chromatography tandem mass‐spectrometry (LC–MS/MS) using a common calibrator. Use of VBCDs was evaluated qualitatively by patients. Mean bias between plasma and volumetrically collected capillary DBS Phe was −13%. Mean recovery (SD) of Phe from DBS was 89.4% (4.6). VBCDs confirmed that the volume of blood typically assumed to be present in a 3.2 mm sub‐punch is over‐estimated by 9.7%. Determination of the relationship between plasma and capillary DBS Phe, using a single analytical method, common calibration and VBCDs, demonstrated that once the under‐recovery of Phe from DBS has been taken into account, there is no significant difference in the concentration of Phe in plasma and capillary blood. Conversely, comparison of plasma Phe with capillary DBS Phe collected on a NBS card highlighted the limitations of this approach. Introducing VBCDs for the routine monitoring of patients with PKU would provide a simple, acceptable specimen collection technique that ensures consistent sample quality and produces accurate and precise blood Phe results which are interchangeable with plasma Phe.

**Synopsis**

Volumetric blood collection devices provide a simple, acceptable specimen collection technique that ensures consistent sample quality and produces accurate and precise blood Phe results which are interchangeable with plasma Phe.

## 1 INTRODUCTION

Accurate and reproducible measurement of phenylalanine (Phe) is key to the management of patients with phenylketonuria (PKU, OMIM 261600); the 2017 European guidelines for diagnosis and management of patients with PKU recommend consensus age‐related blood phenylalanine (Phe) target treatment ranges to prevent adverse neurological outcomes and specify three clinical decision points (120, 360 and 600 μmol/L).^1^ Sapropterin responsiveness is defined by a decrease in blood Phe ≥30%.^2^, ^3^ However, the guidelines simply refer to ‘blood Phe’ throughout, without differentiating between specimen type (venous/capillary blood, plasma and dried blood spots (DBS)). Given that DBS Phe concentrations are generally considered to be lower than plasma, the reported bias varying from 15% to 28%,^4^, ^5^, ^6^, ^7^, ^8^ the absence of detailed information on the specimen type used in the studies from which the guidelines were derived is an oversight. Furthermore, the evidence was derived from multiple studies which utilised different methods of analysis and different specimen types. Although the guidelines state that ‘Phe measurements should be robust’ and that ‘the accuracy of ion exchange analysers (IEC) analysers, high performance liquid chromatography (HPLC) and mass spectrometry (MS) is well established’,^1^ accuracy reflects standardisation and calibration as well as the selectivity of the methodology itself. Table 1 summarises the methodology and specimen type used during the studies from which the treatment guidelines were derived. The approach to calibration was not provided for any of these studies.^9^, ^10^, ^11^, ^12^, ^13^, ^14^, ^15^, ^16^, ^17^, ^18^, ^19^, ^20^, ^21^, ^22^, ^23^, ^24^

**TABLE 1:** Summary of the methodology and sample type used in the studies from which the evidence base for the 2017 European guidelines for the diagnosis and management of patients with phenylketonuria were derived.

Study	Method details	Specimen type
Weglage et al.^9^	Combination of NBS, lifetime Phe and study Phe results used. Study Phe measured using HPLC (method of detection not stated). NBS and lifetime Phe methods not stated.	Serum during study. NBS and lifetime Phe specimen type not stated
Bick et al.^10^	Not stated	Plasma
Kono et al.^11^	Not stated	Serum
Cleary et al.^12^	HPLC (lithium cation exchange column), or TLC (method of detection not stated)	Blood
Manara et al.^13^	Tandem MS/MS	Plasma and DBS
Waisbren et al.^14^	Meta‐analysis (variety of methods included: chromatographic, enzymatic and fluorometric). Authors made the assumption that the analytical methods used in the labs yielded equivalent results.	Blood
Fonnesbeck et al.^15^	Meta‐analysis (does not specify which methods or sample types are included)	Blood
Weglage et al.^16^	HPLC (method of detection not stated)	Blood
Diamond et al.^17^	Measured by two different labs. One lab measured Phe by HPLC (method of detection not stated). The other lab measured Phe by the Guthrie test when Phe <10 mg/dL and by HPLC (method of detection not stated) or McCaman–Robins fluometric assay when Phe concentrations approached 10 mg/dL.	Plasma
Leuzzi et al.^18^	Not stated	Blood
Huijbregts et al.^19^	Not stated	Not stated
Schmidt et al.^20^	Not stated	Not stated
Jahja et al.^21^	Lifetime Phe concentration calculated from historical Phe results, method not stated for either concurrent or lifetime Phe.	Blood
ten Hoedt et al.^22^	Tandem MS/MS (neutral loss)	DBS
Hoeksma et al.^23^	HPLC (Waters AccQ Tag method) (method of detection not stated)	Plasma
Sanayama et al.^24^	Not stated	Uses ‘serum’ and ‘plasma’ Phe interchangeably throughout

Data from the 2022 European Research Network for the evaluation and improvement of screening, Diagnosis and treatment of Inherited disorders of Metabolism (ERNDIM) Quantitative Amino Acids External Quality Assessment (EQA) scheme and Special Assays in Dried Blood Spots (SADBS) EQA scheme show that the mean inter‐laboratory relative standard deviation (%RSD) for Phe in plasma (*n* = 310) and DBS (*n* = 95) was 9.8% and 20.1% respectively.^25^ For the SADBS scheme, mean intra‐laboratory imprecision was 9.0%, demonstrating accuracy is a significant contributor to inter‐laboratory variation and highlighting a limitation of this scheme; participant results are scored relative to the all‐laboratory trimmed mean and do not reflect the target/true value.

The aim of this study was to compare Phe concentrations in plasma, with blood collected using the Capitainer‐qDBS volumetric blood collection device (VBCD) and the non‐volumetric conventional NBS collection card routinely used for PKU monitoring. Analysis by LC–MS/MS and quantification against a common calibrant compensated for measurement bias due to methodology, calibration or standardisation. Likewise, the use of VBCDs negated differences due to DBS specimen quality, volume and haematocrit of the blood applied to the filter paper and/or assumed volume of blood in a sub‐punch from a DBS specimen.^26^

## 2 MATERIALS AND METHODS

**FIGURE 1:**
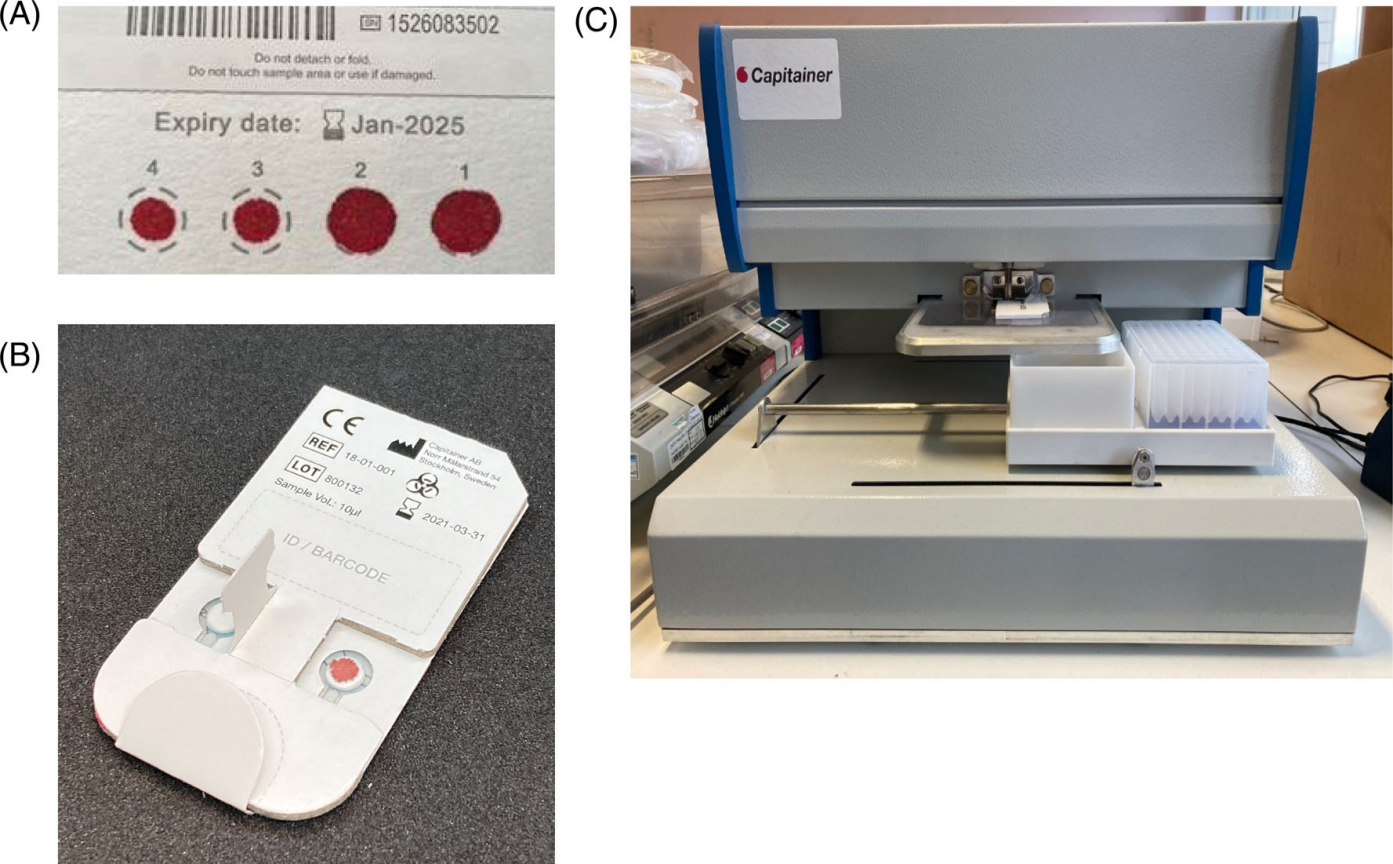
Images of (A) the conventional PerkinElmer‐226 filter paper collection device, (B) the Capitainer‐qDBS volumetric blood collection device and (C) the semi‐automated disc removal tool.

Full details of the materials and methods used are provided in Appendix S1. Images of the collection devices and disc removal tool (DRT) are shown in Figure 1A–C respectively.

### 2.1 Patient samples

The study was reviewed by local research and development departments and approved as a service evaluation; formal ethical approval was not required. Patients (*n* = 51) were provided with information sheets and gave informed consent. Three paired samples were collected from each patient during a routine clinic visit; venous blood (5 mL lithium heparin); capillary blood on a Capitainer‐qDBS VBCD (2× 10 μL); capillary blood on a filter paper collection device (2 drops). Immediately post‐collection the venous blood was centrifuged and plasma removed and stored at −20°C until analysis. Capillary blood samples were dried at ambient temperature for 3 h then stored in foil bags with desiccant at −20°C. The quality of the DBS specimens was assessed against the proposed UK Metabolic Biochemistry Network guidelines for monitoring of inherited metabolic diseases.^27^ All samples met the acceptance criteria. Images of the collection devices and DRT are shown in Figure 1A–C respectively.

### 2.2 Qualitative evaluation of the Capitainer‐qDBS

A questionnaire was distributed to *n* = 37 patients with PKU at the 2022 National Society for Phenylketonuria annual symposium. The questionnaire was designed to evaluate satisfaction with the device in terms of; ease of use; clarity of instructions; ease with which blood could be applied; comparison with conventional NBS filter paper collection. Questions were scored on a scale of 1–10, with 1 indicating a negative response and 10 indicating a positive response. A copy of the questionnaire can be found in Appendix S2.

## 3 RESULTS

A detailed summary of the analytical performance parameters can be found in Appendix S1.

**FIGURE 2:**
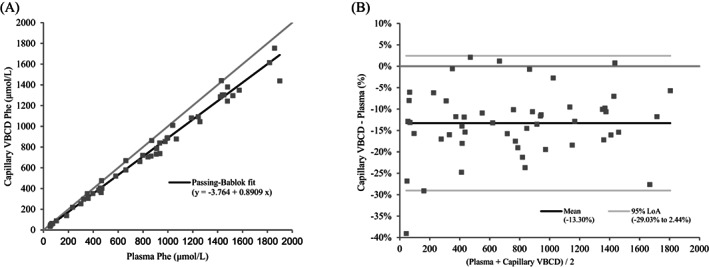
(A) Passing–Bablok regression demonstrating the correlation between plasma phenylalanine and capillary phenylalanine collected with a volumetric blood collection device. (B) Bland–Altman analysis demonstrating the agreement between plasma phenylalanine and capillary phenylalanine collected with a volumetric blood collection device.

Plasma Phe concentrations in the patient specimens ranged from 52 to 1899 μmol/L (mean 808 μmol/L, median 839 μmol/L). Passing–Bablok regression equation for plasma versus capillary phenylalanine collected with VBCDs was *y* = −3.764 + 0.8909*x*, *R* ^2^ 0.992 (*n* = 51). The 95% confidence intervals (CI) for the slope and intercept were 0.8598–0.9130 and −17.31 to 5.042 respectively, indicating a proportional difference between plasma and capillary Phe but no systematic error (Figure 2A). The cusum test confirmed no significant deviation from linearity (*p* = 0.9889) and the residual standard deviation (95% CI) was 45.5 (−89.2 to 89.2). Bland–Altman analysis showed a mean bias of −13.3% (95% limits of agreement (LoA) −29.03 to 2.44%) (Figure 2B). Passing–Bablok regression equation for capillary Phe collected volumetrically versus non‐volumetrically was *y* = −0.09517 + 1.091*x*, *R* ^2^ 0.975 (*n* = 52). The 95% CI for the slope and intercept were 1.019–1.178 and −20.15 to 19.31 respectively, indicating a proportional difference between volumetric and non‐volumetric capillary DBS but no systematic error. The cusum test confirmed no significant deviation from linearity (*p* = 0.6862). The RSD (±1.96 RSD interval) was 71.61083 (−140.3522 to 140.3522). Bland–Altman analysis showed a mean bias of 9.42% (95% LoA −14.32 to 33.17%).

**FIGURE 3:**
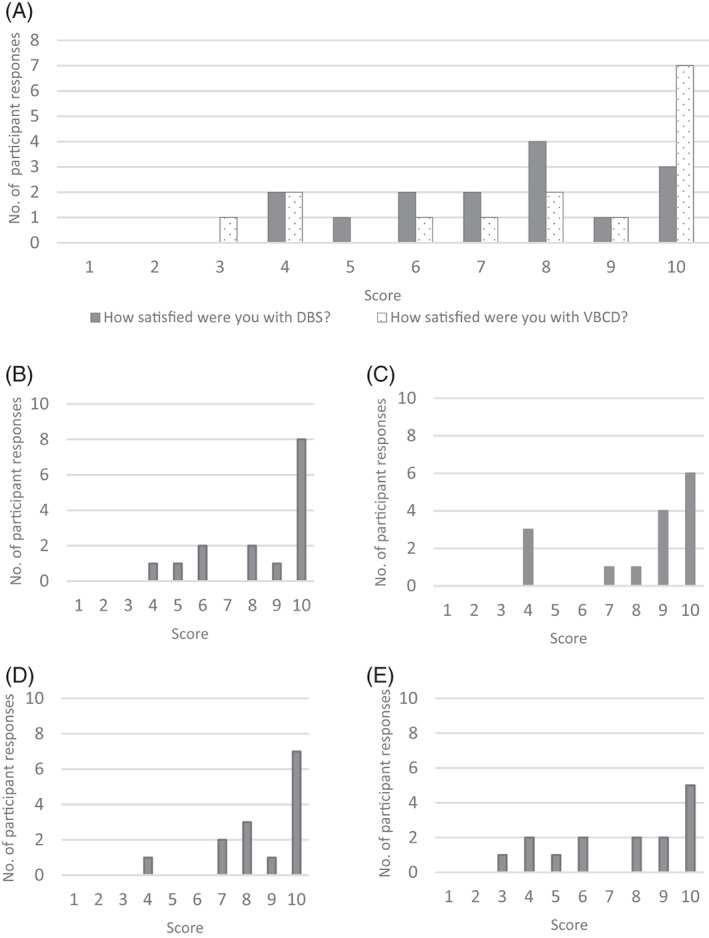
Summary of responses to the qualitative evaluation of the Capitainer‐qDBS volumetric blood collection device summarising responses to the following questions: (A) overall satisfaction with sampling method compared with the standard NBS collection card; (B) ease of detecting the colour change indicating successful sampling; (C) ease of use compared with the standard NBS collection card; (D) how easy it was to follow the Capitainer‐qDBS sampling instructions; and (E) ease with which blood could be applied to the Capitainer‐qDBS device. Responses to each question were scored on a scale of 1 = ‘very difficult’ to 10 = ‘very easy’ apart from (A) where the scale was 1 = ‘not satisfied’ to 10 = ‘very satisfied’.

The results of the qualitative evaluation of patient satisfaction with the Capitainer‐qDBS are summarised in Figure 3. Questionnaires were returned by 15/37 participants. Feedback was predominantly positive with a mean score of 8/10 for all questions. A copy of the questionnaire results can be found in Appendix S3.

## 4 DISCUSSION

Although traditionally imprecision is often deemed acceptable when <15%,^28^ it should be assessed in relation to the clinical utility of the test in question and its contribution to the total error (TE) of the test.^6^ Using this approach, acceptable test imprecision for Phe has been deemed to be <4.7%,^29^ meaning that the imprecision of both plasma Phe and non‐volumetrically collected blood Phe was slightly higher than acceptable. Whilst this degree of imprecision was expected for the conventional filter paper collection device, plasma Phe assays generally perform better.^6^ However, it is postulated that the greater imprecision seen for DBS reflects the limitations of manually pipetting a smaller sample volume (10 μL) than the larger volume (e.g., 50 μL) used in IEC or HPLC assays. In addition, the random error (scatter) associated with non‐volumetrically collected blood Phe is significantly greater than that seen with the VBCD, as evidenced by the increase in residual standard deviation when comparing the correlation of plasma Phe to capillary Phe measured with these devices (45.5 vs. 71.6 for VBCD and conventional NBS collection device respectively). The superior precision of the VBCD is advantageous for long‐term monitoring of individual patients as it minimises the TE associated with the measurement of Phe.

The results show that the mean bias between plasma and capillary blood Phe using VBCDs is −13%. The use of a common analytical method, calibration and operator excludes method and/or calibration bias as contributing to this difference, a key differentiator from previously reported studies.^4^, ^5^, ^6^, ^7^, ^8^, ^30^, ^31^ Likewise, the use of the VBCDs rather than conventional NBS cards, negates for differences due to specimen quality, blood haematocrit and/or volume of blood applied to the collection card. It also avoids the need to make assumptions about the volume of blood present in the sub‐punch, which is directly relevant when results are quantified by internal calibration. One explanation for this apparent bias is under‐recovery of Phe from DBS specimens. Assuming that recovery of Phe from plasma equals 100%, the −13% bias noted between plasma and DBS Phe in this study could be accounted for, within experimental error, by the under‐recovery of Phe from DBS (89%), although it should be noted that a limitation of this hypothesis is that the recovery data relates to Ahlstrom‐226 filter paper, whereas the bias was relative to Ahlstrom‐222 paper. If the VBCD Phe results are corrected for the under‐recovery, the relationship between plasma and capillary DBS is *y* = 0.9981*x* + 3.0304, *R* ^2^ = 0.9838.

An alternative explanation is that there are differences in the distribution of Phe between plasma and erythrocytes/blood. However, it has been demonstrated previously^32^ that there is no difference in the concentration of Phe between plasma and blood when experimental error is taken into account: mean plasma Phe 53 ± 8 μmol/L and mean blood Phe 49 ± 7 μmol/L (*n* = 20).

Establishing that there is no physiological difference between plasma and capillary DBS Phe means laboratories can routinely use an aqueous calibration to measure both plasma and capillary DBS Phe, provided they account for the recovery of Phe from the DBS and collect the specimens with a VBCD. This is advantageous as it provides a common point of reference within a given laboratory and importantly, between laboratories; a certified reference material for Phe in aqueous solution being commercially available (*Trace*CERT®). By implementing these changes, laboratories could prevent misinterpretation of Phe concentrations derived from different specimen types. This would be especially useful in paediatric and pregnant patients, where treatment targets have been linked explicitly with clinical outcomes such as neurological function in paediatric patients, and maternal PKU syndrome in pregnant individuals respectively.^1^ In both instances, the difference in Phe concentration between “in‐control” and “sub‐optimal control” is separated by a narrow margin, the magnitude of which is similar to the previously reported differences between specimen types.^6^

This study highlights the limitations of monitoring DBS Phe with conventional, non‐volumetric NBS cards and the impact this has when establishing the relationship between Phe in plasma and DBS. For laboratories that quantify DBS Phe using internal calibration, the impact is even greater since the volume of blood assumed to be in the DBS sub‐punch is included in the calculation of the measured Phe concentration. For example, if the volume of blood present in a 3.2 mm sub‐punch is assumed to be 3.1 μL, based on an assay where the sub‐punch is eluted with 150 μL of eluent and Phe results are quantified by internal calibration, the final concentration of Phe would be calculated as follows: Phe (μmol/L) = (analyte response/SIL response) × (concentration of SIL) × (150/3.1). Hence, if the volume of blood in a 3.2 mm sub‐punch was overestimated, and is in fact 2.8 μL, the error introduced would result in the “true” Phe concentration being underestimated, that is, the difference between (150/3.1) and (150/2.8) equates to (53.57–48.39)/53.57, equivalent to a difference of −9.7%.

Determining the exact volume of blood present in a 3.2 mm sub‐punch of a conventional DBS is not the intended aim of this study. Experimental determination of this parameter is complex^33^, ^34^, ^35^ and several factors contribute directly to the final volume of blood in a sub‐punch of fixed diameter; volume of blood from which the DBS is formed; bloodspot size; punch size and location; blood haematocrit and choice of filter paper.^26^ Therefore, unless each of these factors is controlled, which is not possible with clinical specimens, the exact volume of blood in a sub‐punch cannot be accurately defined. The authors' intention is to raise awareness of the limitations of non‐volumetrically collected capillary blood specimens and highlight that the use of VBCDs enables the collection of an accurate and precise volume of blood whilst ensuring the quality of the DBS collected. This is important because clinical laboratories must adhere to ISO:15189:2012 standards^36^ which specify that ‘the laboratory shall have information available for patients and users of the laboratory services’. The information shall include as appropriate; the laboratory's criteria for accepting and rejecting samples; a list of factors known to significantly affect the performance of the examination or the interpretation of the results. If a sample does not meet the laboratory developed and documented criteria for acceptance, it should be rejected. The historic practice where laboratories have employed a more lenient approach to ‘special’ specimens is no longer acceptable, yet many laboratories remain reluctant to reject DBS specimens, particularly from children, which leads to considerable variation in practice. A recent audit^27^ demonstrated that introducing DBS quality acceptance criteria would increase specimen rejection rates in England from 4% to 22%. Extrapolating this to the annual Phe monitoring workload in England would correspond to the rejection of 12 410 specimens each year.

Introducing VBCDs would potentially solve this problem and, whilst it is acknowledged that the Captainer qDBS was evaluated by a small number of patients, the results indicate that the device would likely be well received by this patient group. However, the device costs approximately £2.50 more than the conventional NBS card. Based on the number of patients on diet in England, the recommended frequency of testing^37^ and accounting for a 22% rejection rate with the conventional NBS card, introducing the Capitainer‐qDBS would increase costs by approximately £124 K, or £68 per patient, per year. Consideration should be given to the financial impact of implementing these devices, balanced against the benefits of more accurate and precise results, reducing anxiety and/or minimising the potential harm associated with repeat requests.

## 5 CONCLUSION

Implementing VBCDs for the routine monitoring of blood Phe will ensure better quality specimens by negating issues relating to DBS size, quality and haematocrit. This should be considered especially in patient subgroups where improved precision and accuracy of results, and avoidance of sample rejection could improve clinical care such as in children, pregnant woman and during sapropterin responsiveness testing. This study emphasises the need for clinical and laboratory health care professionals to better understand the true relationship between plasma and capillary DBS Phe, acknowledge the limitations of the current methodologies and translate this into clinical practice to ensure correct interpretation of laboratory tests and appropriate patient management. It would be advantageous to revise the 2017 guidelines using evidence from studies where appropriate scrutiny has been given to the analytical performance of the method used. The potential application of VBCDs to monitor other inherited metabolic disorders, for example, tyrosinemia type 1, maple syrup urine disease or classical homocystinuria, should also be investigated.

## AUTHOR CONTRIBUTIONS

**Rachel S. Carling**: conceptualisation, methodology, formal analysis, supervision, writing—original draft preparation. **Zoe Barclay**: investigation, validation, formal analysis, writing—review and editing. **Nathan Cantley**, **Sarah L. Hogg**, **Yael Finezilber**, **Elaine Murphy**, and **Danja Schulenburg‐Brand**: resources, writing—review and editing. **Erin C. Emmett**: writing—review and editing. **Stuart J. Moat**: validation, writing—original draft preparation.

## CONFLICT OF INTEREST STATEMENT

Rachel Carling, Zoe Barclay, Nathan Cantley, Erin Emmett, Sarah Hogg, Yael Finezilber, Danja Schulenburg‐Brand, Laine Murphy and Stuart Moat declare that they have no conflict of interest.

## INFORMED CONSENT

All procedures followed were in accordance with the ethical standards of the responsible committee on human experimentation (institutional and national) and with the Helsinki Declaration of 1975, as revised in 2000 (5). Informed consent was obtained from all patients for being included in the study.

## Supporting information


**Appendix S1:** Supplementary Information

**Appendix S2:** Supplementary Information

**Appendix S3:** Supplementary Information

## Data Availability

The data that support the findings of this study are available from the corresponding author upon reasonable request.
