# Supplementary material for: Investigation of the relationship between phenylalanine in venous plasma and capillary blood using volumetric blood collection devices
Source: JIMD Rep. 2023 Oct 16;64(6):468–76. doi: 10.1002/jmd2.12398 (PMC10623100; doi:10.1002/jmd2.12398)
Supplement: Supplementary file 1 — Appendix S1: Supplementary Information [file JMD2-64-468-s003.docx]

**Supplementary material S1**.

**Chemicals and reagents**

LC-MS grade methanol, formic acid and water were from Fisher Scientific (Loughborough, UK). L-phenylalanine (>98%) and Amino acids mix solution, *Trace*CERT®, were from Sigma Aldrich (Dorset, UK). L-phenylalanine (ring-D5, 98%) was from CK Isotopes (Leicestershire, UK). Phe stable isotope label (SIL) stock standard (50mL, 3mM) and Phe stock standard (50mL, 25mM) were prepared in distilled water containing HCl (1M, 200µL). Extraction eluent (12µM) was prepared by addition of SIL stock (800µL) to 80% methanol (200mL).

Capitainer-qDBS devices and the semi-automated disc removal tool (DRT) were obtained from Capitainer (Solna, Sweden).^1^ Conventional filter paper collection devices, incorporating Ahlstrom-226 filter paper, (nominal thickness 0.54 mm) used in the English NBS programme were obtained from CDP Print Management (Uxbridge, England). Images of the collection devices and DRT are shown in Figures 1a-c respectively.

A 7-plus-blank calibration curve was prepared by dilution of the Phe stock standard with water to give final concentrations of 0, 15, 52, 202, 404, 606, 909 and 1515 µM. Accuracy of the calibration was verified against the certified reference material (*Trace*CERT®).

**Preparation of internal quality controls**

Blood was collected from healthy volunteers into lithium heparin tubes (Greiner Bio-One, Gloucestershire, UK). Informed consent was obtained in-line with local governance arrangements. Endogenous Phe concentrations were measured using a modification of a multiplexed LC-MS/MS method described previously^2^. Blood (60mL) was split into three portions. One pool was used at endogenous concentration, the others were enriched with Phe to final concentrations of 374 and 758 µmol/L. Volumetric DBS specimens were prepared by application of blood (10µL) to pre-perforated Ahlstrom-222 filter paper with a positive displacement pipette. Blood spots were dried at ambient temperature for 3 hours then stored in foil bags with desiccant at -20°C. The imprecision of volumetric DBS specimens collected on the Capitainer qDBS device has been established previously^3^ (mean %RSD 2.1) and was not significantly different to the imprecision seen when volumetric DBS were prepared on pre-perforated Ahlstrom-222 paper (p>0.01) so preparing IQC materials on Ahlstrom -226 paper was a more cost effective approach. Non-volumetric DBS specimens were prepared by application of blood (50µL) to Ahlstrom-226 filter paper with a positive displacement pipette. Blood spots were dried at ambient temperature for 3 hours then stored in foil bags with desiccant at -20°C. Plasma samples were prepared by centrifuging the blood for 10 mins (3000 rpm). Plasma was removed and aliquots (100µL) were stored at -20°C.

Additional samples were prepared in an identical manner to assess recovery of Phe from DBS. Blood (25mL) was split into five equal portions. One pool was used at endogenous concentration, the others were enriched with Phe at concentrations equivalent to 250, 500, 800 and 1200µmol/L. Volumetric DBS specimens were prepared by application of blood (10µL) to Ahlstrom-226 filter paper with a positive displacement pipette) . Blood spots were dried at ambient temperature for 3 hours then stored in foil bags with desiccant at -20°C. Recovery was assessed relative to paired aqueous samples prepared in parallel.

**Sample preparation**

Calibrators, plasma samples and IQC (10µL) were added to a 96 square-well (2mL) collection plate (Waters, Wilmslow, UK) with a Gilson pipette. For patient samples collected with VBCDs, the 6mm filter-paper disc was removed from the device and placed into the 96-square-well plate by the semi-automated disc removal tool (Figure 1c). For volumetric (10µL) DBS IQC materials prepared in-house, the entire DBS specimen was removed with a handheld cutting tool, 6mm diameter, and placed into the 96 square-well plate. For non-volumetric DBS specimens (patient/IQC) a 3.2mm sub-punch was removed from the centre of each specimen with a Wallac DBS puncher (PerkinElmer, Turku, Finland) and placed directly into the 96-square-well plate.

Extraction eluent (300µL) was added to each sample in the 96-square-well plate. Samples were agitated on an Eppendorf Thermomixer ® C (1800 rpm, 35 minutes) at ambient temperature. Sample (50µL) was transferred to a clean 96-deep-well plate and diluted five-fold with 80% methanol.

**Analysis of samples by liquid chromatography tandem mass spectrometry (LC-MS/MS)**

Sample (1µL) was injected into an ABSciex 4500 MS with electrospray ionisation source coupled to a Shimadzu ExionLC™ system with a Waters Cortecs T3 column (2.1 x 50mm, 2.7 µm) held at 55°C. Mobile phase A was water with 0.1% formic acid, 10mM ammonium acetate. Mobile phase B was methanol with 0.1% formic acid, 10mM ammonium acetate. Flow rate was 600 µL/min. Initial conditions were 98% A, changing to 2% between 0.6 and 0.65 minutes, holding until 1.0 minute, returning to 98% A by 1.01 minutes then re-equilibrating for 0.59 minutes. Instrument settings were curtain gas 35 psi, collision gas 8, ion spray voltage 5500 V, temperature 500 ºC, ion source gas 1, 60 psi and ion source gas 2, 40 psi. Cone gas and collision energy were optimised for each analyte. Data were acquired by selective reaction monitoring using positive ionisation mode (Phe 166>120). The method described is in routine use at the author’s (RSC’s) laboratory and is accredited to ISO 15189:2012 standard.

Samples were quantified by stable isotope dilution against the aqueous calibration curve. For the non-volumetric samples, the volume of blood present in the 3.2mm sub-punch was assumed to be 3.1µL,^4^ consistent with current practice in the UK. To correct for the volume difference compared to the aqueous calibrators (10µL), a correction factor of 3.226 was applied i.e., 10/3.1. The volume of blood present in the sub-punch was also back calculated using the measured concentration of Phe in the 10µL VBCD as the true value.

**Statistical Analysis**

Inter-assay imprecision (%RSD) was determined by replicate measurements (n=25) over five days of two IQC materials for both plasma and dried blood. Acceptable test imprecision for Phe is deemed to be <4.7%.^5-6^ An f-test was used to determine if there was significant difference in variance (*p*<0.01). Passing–Bablok regression was used to determine whether there was correlation between the different sample sets. The assumption of linearity was tested using the cusum test (*p* value < 0.05 indicating significant deviation from linearity). Random difference between the sample sets was assessed using the residual standard deviation (RSD) with 95% of random differences expected to lie within the interval -1.96 to +1.96 RSD. Bland–Altman plots were used to evaluate the agreement between the different sample sets. Statistical analysis was performed with Microsoft-Excel 2016 and Analyse-it (Version 4.95).

**Results**

Inter-assay imprecision of plasma Phe was unacceptable (mean RSD of IQC was 6.9%). Inter-assay imprecision of non-volumetrically collected DBS Phe was also unacceptable (mean RSD of IQC was 6.2%). Inter-assay imprecision of volumetrically collected DBS Phe was acceptable (mean RSD of IQCs was 2.6%). An f-test demonstrated that volumetrically prepared DBS were significantly more precise than either plasma or conventional DBS (*p* <0.01). Mean recovery of Phe from DBS (n=15) was 89.4% (SD 4.6, range 82.5 – 96.3%).

The mean (SD) volume of blood present in a 3.2mm sub-punch taken from a DBS formed from 50µL blood was 2.8 (0.3)µL, (range 2.0-3.5). The current assumption in the UK (3.1µL) does not account for the volume of blood from which the specimen is formed, thus it over-estimates the volume of blood present in a 3.2mm sub-punch. This results in the Phe concentration of a 3.2mm sub-punch quantified against a 10µL calibrator being under-estimated by 9.7%, broadly consistent with the relationship described above.

**References**

1. Velghe S, Stove C (2018) Evaluation of the capitainer-B microfluidic device as a new hematocrit-independent alternative for dried blood spot collection. Anal Chem. 90: 12893-9.
2. Carling RS, John K, Churchus R, Turner C, Dalton NR (2020) Validation of a rapid, comprehensive and clinically relevant amino acid profile by underivatised liquid chromatography tandem mass spectrometry. Clin Chem Lab Med.58(5):758-768
3. Carling RS, Emmett EC, Moat SJ. (2022) Evaluation of volumetric blood collection devices for the measurement of phenylalanine and tyrosine to monitor patients with phenylketonuria. Clin Chim Acta 535: 157-166. doi: 10.1016/j.cca.2022.08.005
4. Adam B, Alexander J, Smith S, Chace D, Loeber J, Elvers L, et al. (2000) Recoveries of phenylalanine from two sets of DriedBlood-spot reference materials: Prediction from hematocrit, spot volume, and paper matrix. Clin Chem 46(1): 126-128.
5. Corte Z, Venta R (2010) Biological variation of free plasma amino acids in healthy individuals. Clin Chem Lab Med 48: 99-104.
6. Moat S, Schulenburg-Brand D, Lemonde H, Bonham J, Weykamp C, Mei J, et al. (2019) Performance of laboratory tests used to measure blood phenylalanine for the monitoring of patients with phenylketonuria. J Inherit Metab Dis 43(2): 1-10.
