# Supplementary material for: Investigation of the relationship between phenylalanine in venous plasma and capillary blood using volumetric blood collection devices
Source: JIMD Rep. 2023 Oct 16;64(6):468–76. doi: 10.1002/jmd2.12398 (PMC10623100; doi:10.1002/jmd2.12398)
Supplement: Supplementary file 2 — Appendix S2: Supplementary Information [file JMD2-64-468-s002.pdf]

Service evaluation – blood collecting devices

For each question please select the most appropriate answer:

1. Please state the age group of the person taking the sample:

   ☐ under 17
   ☐ over 17

2. Are you taking the sample

   ☐ for yourself?
   ☐ on behalf of someone else?

   If someone else, please state their age: ________

3. What is your reason for sending bloodspot samples?

   ☐ PKU monitoring
   ☐ Tyrosinaemia monitoring
   ☐ other (please specify):

   ____

4. How often do you take bloodspot samples?

   ☐ daily
   ☐ biweekly
   ☐ weekly
   ☐ monthly
   ☐ annually

5. How long have you been taking bloodspot samples for? ________

6. On how many occasions have you used the Capitainer device?

   ☐ once
   ☐ less than 5 times
   ☐ more than 5 times

Instructions

7. How easy did you find the instructions for the Capitainer to follow?

Very difficult	1	2	3	4	5	6	7	8	9	10	Very easy

8. Were you provided with training prior to collection? Yes No

9. Were you able to understand the language used? Yes No

10. After receiving instruction how confident did you feel to use the device?

Not at all confident	1	2	3	4	5	6	7	8	9	10	Very confident

Use

1. How easy did you find the Capitainer to use compared to a bloodspot (Guthrie) card?

Very difficult	1	2	3	4	5	6	7	8	9	10	Very easy

2. How easy was it to apply blood to the Capitainer device?

Very difficult	1	2	3	4	5	6	7	8	9	10	Very easy

3. How easily detectable was the colour change?

Very difficult	1	2	3	4	5	6	7	8	9	10	Very easy

Summary

4. Overall, how satisfied were you with each sampling method?

Bloodspot card

Not satisfied	1	2	3	4	5	6	7	8	9	10	Satisfied

Capitainer Device

Not satisfied	1	2	3	4	5	6	7	8	9	10	Satisfied

Any other comments?
